# Mechanisms of change in a gender-sensitized health intervention: the mediating role of health self-efficacy

**DOI:** 10.1186/s40359-025-02658-4

**Published:** 2025-04-09

**Authors:** Jeremy S. Vassallo, Kim M. Shearson, Jenny M. Sharples

**Affiliations:** Institute for Health and Sport, Victoria University, Footscray Park, Ballarat Rd, Melbourne, VIC Australia

**Keywords:** Health behavior change, Gender-sensitised intervention, Men’s health, Self-efficacy, Masculinity

## Abstract

**Background:**

Men’s lower life expectancy has been in part attributed to adherence to masculine stereotypes restricting health promoting behaviors. Health-specific self-efficacy beliefs are theorised to contribute to these outcomes and recent research suggests addressing the link between masculinity and these beliefs is a key pathway to men’s health promotion. Gender-sensitised health interventions show promise for helping men overcome health barriers as the “safe space” provides opportunity to broaden perspectives of masculinity, contributing to increases in health self-efficacy, which then mediates improved health outcomes. This study aimed to address gaps in previous research by examining this mechanism of gender-sensitised health promotion longitudinally in an intervention context.

**Methods:**

A sample of 295 men participating in the Sons of the West (SOTW) 2019 program, a 10-week gender-sensitised, community-based health program, were measured pre-program and post-program to longitudinally assess changes in health outcomes. Regression analyses were used to test a mediation model of health behavior change.

**Results:**

Findings showed improvements in health behavior, health self-efficacy, psychological distress, and decreased conformity to masculine norms. Residualized scores of the changes from pre-intervention to post-intervention indicated the changes in health self-efficacy fully mediated the relationship between changes in conformity to masculine norms and changes in health behavior and psychological distress.

**Conclusions:**

Results suggest the key mechanism of change was the provision of opportunities for men to negotiate masculine stereotypes thus increasing health self-efficacy and explaining health improvements. This study utilized data from a real-world, large-scale men’s health program but was limited to two waves of data. Future research and implications are discussed.

## Background

Men’s life expectancy is in general lower than women’s; in Australia, there is a six-year difference in median age at death [1]. Men’s life expectancy is further reduced according to the socioeconomic area in which they live; in the year this study was conducted, men’s median age of death was 4 years less in the lowest quintile compared to the highest [1]. Higher mortality in men is in part attributed to men’s perceptions of health promotion and their reluctance to undertake preventive behaviors, which is linked to men’s conformity to masculine norms [2]. Traditional western masculinity promotes the ideal of the strong, stoic, independent man associated with men’s perceptions that seeking help shows weakness, regular check-ups are unnecessary unless seriously ill, and that health problems should be endured rather than treated [3, 4]. Masculine norms are defined as socially constructed beliefs and behaviors of men, adherence to which is considered necessary to maintain social status, power and meet societal expectations [5]. Empirical literature suggests masculinity entails several distinct beliefs related to emotional restriction, self-reliance, and aggression [6]. Mahalik et al. [6] found those on the higher end of the masculinity continuum report excessive risk-taking behavior, and alcohol and tobacco use. Conformity to masculine norms is a multidimensional concept that encompasses the gamut of western-specific norms and consistently predicts negative health related outcomes such as fewer doctor visits, less engagement with a mental health professional, and higher substance use [7]. Furthermore, a recent meta-analysis [8] found many subdomains of masculinity predict negative health outcomes, with self-reliance routinely the strongest for poor mental health outcomes compared to other subdomains. In contrast, Gerdes and Levant’s [7] content analysis of 17 studies found positive associations between some masculinity subdomains and health outcomes in 30% of the findings reviewed, particularly those related to health promotion. Similarly, Sagano et al. [9] found that whilst global conformity to masculine norms was negatively associated with health behaviours, some specific subdomains (emotional control and focus on success and winning) were associated with protective health behaviors and others (risk-taking, power over women, self-reliance and endorsing casual sexual activity) with health-risk behaviors, suggesting a complex interplay between masculinity and health outcomes.

Moreover, findings from a large representative sample of Australian men found that health service use was positively associated with some aspects of masculinity and negatively predicted by others [10]. Furthermore, the salience of masculinity sub-domains varied across generational age cohorts. The authors argued a gender-sensitized approach that includes age appropriate interventions is needed to improve men’s health. Men of lower socio-economic status face greater health disparities not only due to lack of health care access and opportunities to engage in health prevention related to social determinants of health [11], but because negative perceptions about preventative health behavior are particularly prevalent among men from lower socioeconomic backgrounds where traditional masculine norms may be more strongly endorsed [4]. Accordingly, Robertson [4] suggests that men’s health interventions must be understood within the wider context of gender relations and social structures, and suggests community-based settings may serve as alternatives to clinical environments by creating a space where men can explore health behaviors in ways that align with, rather than challenge, their masculine identities.

### Masculine norms and self-efficacy

Health self-efficacy beliefs, defined as an individual’s belief in their capacity to carry out a health behavior [12], are instrumental for short-term and long-term health behavior change [13]. A recent meta-analysis [14] shows self-efficacy as the strongest predictor for health-related intentions and behaviors with overall medium effect sizes across 151 studies. The relationship between masculinity and self-efficacy is complex as general self-efficacy has been found to positively correlate with masculinity [15], yet health-specific self-efficacy beliefs negatively correlate [16, 17]. Health self-efficacy beliefs are often task-specific, such as belief in one’s ability to seek health care in a timely manner or confidence in finding enjoyable exercise activities, and it is suggested that many beliefs are defined through gender-role socialization [18]. More specifically, sanctions against health behaviors not deemed typically masculine lead to fewer opportunities to observe and practice health promoting behaviors [19]. Modelling behavior is central to self-efficacy development and may explain the limited development in health behaviors through a lack of role-models [13]. For instance, increased help-seeking behavior and decreased conformity to masculine norms were reported in men after viewing a documentary promoting psychological help-seeking [20]. The authors [20] suggest modelling help-seeking behavior encouraged men to overcome barriers related to self-reliance and consequently increased psychological help-seeking.

General health self-efficacy has been found to mediate the relationship between masculinity and health behaviors [21], and to moderate the relationship between masculinity and psychological helpseeking [22] and perceived barriers to healthcare [23]. However, the role of health specific self-efficacy in the complex relationship between masculinity and men’s health is largely untested. In a community sample, Lease et al. [16] found greater endorsement of masculine norms was associated with lower health self-efficacy, which also predicted poorer health outcomes. They found health self-efficacy mediated the relationship between conformity to masculine norms and health outcomes. This study was limited to a cross-sectional design and did not consider the complex interplay between adaptive and maladaptive aspects of masculinity. The researchers proposed changes in masculine endorsement will lead to increases in health self-efficacy and subsequent health outcomes. However, support for such a hypothesis requires longitudinal data where changes can be observed [24]. Connell and Messerschmidt [25] suggest masculinity is multiple, fluid, and continually co-constructed and change is possible for men who can subvert hegemonic forms of masculinity. This flexibility enables men to practice health promotion behaviors [26]. For instance, when men reframe vulnerability and self-disclosure as brave, the likelihood of psychological help-seeking is increased. Further research is needed to longitudinally examine whether changes in conformity to masculine norms influence changes in health self-efficacy and the degree that this association explains improvements in health outcomes.

### Gender-sensitised health promotion

Men who conform to some masculine norms may see self-care as intrinsically feminine and so neglect health promotion in pursuit of masculine status [27]. Adherence to traditional masculine norms may also foster a reluctance to participate in health and mental health programs as those adhering to traditional masculine norms may similarly see health promotion as feminine [5]. For example, a recent systematic review on mental health intervention research found that on average, men made up 22% of the sample across the reviewed studies [28]. A recent meta-analysis of the effectiveness of men’s health behavior change interventions found that interventions classified as gender-sensitised yielded greater outcomes compared to conventional approaches [29] and research has illustrated their efficacy to engage reluctant men [30, 31]. Gender-sensitised health promotion is defined as tailored approaches to help men overcome barriers to engagement related to adherence to masculine norms [32, 33, 34] and research shows these interventions have moderate to large effects on physical activity, self-esteem, and well-being [31], and on health self-efficacy, adherence to masculine norms, and psychological distress [17]. For example, the Football Fans in Training (FFIT) [35] program demonstrates how gender-sensitised approaches can successfully engage men in health promotion. By situating weight management and lifestyle change within professional football club settings and utilizing community coaches as program leaders, FFIT leverages masculine spaces and identities to overcome traditional barriers to male participation. The program achieved significant results, with participants losing 4.94 kg more than controls at 12 months and showing improvements in physical activity and well-being.

Gender-sensitised approaches embody principals to foster a safe space for participants such as providing a non-threatening environment and male-only participation [17, 27]. Men are often provided with numerous incentives and participation provides esteemed group membership, particularly in programs that utilize the branding of professional sports clubs that are culturally valued. Improvements in men’s health resulting from gender-sensitised interventions are attributed to men’s increased flexibility towards masculinity, which in turn facilitates health promoting behaviours [36, 37, 38]. Recent quantitative research reporting positive changes in self-efficacy and masculine norm adherence through gender-sensitised health promotion holds promise and illuminates a potential mechanism of action [17, 2]. 

## Current study

Gender-sensitised health promotion may be effective as it provides a safe space that sanctions health behaviors sometimes deemed ‘unmasculine.’ Associated reductions in adherence to masculine stereotypes may allow health self-efficacy beliefs to develop. However, this model has only been demonstrated cross-sectionally [16]. Longitudinal research is necessary to address this gap and examine whether increased flexibility towards endorsing traditional masculine norms lead to increases in self-efficacy and associated health benefits. Assessing changes in a gender-sensitized setting that attracts a large sample of men from socioeconomic areas where poor health is evident, provides an opportunity to explore pathways to health improvement. The aim of the current study was to investigate health self-efficacy as a mechanism of change in gender-sensitised health promotion and whether it accounts for the relationship between changes in adherence to masculine norms and health outcomes. First, it was hypothesized improvements in health self-efficacy, health behavior, conformity to masculine norms and psychological distress would be observed across the course of the SOTW program. It was also hypothesized changes in self-efficacy would mediate the relationship between changes in conformity to masculine norms and changes in health behaviors and psychological distress.

## Method

### Program context

Sons of the West (SOTW) is a gender-sensitised health behaviour change program developed over time in response to community needs by the community service arm of the Western Bulldogs Football Club. The program uses the branding of the football club, which is a culturally valued entity in the region, to attract men who might typically avoid more clinically based programs. The program runs once a week over 10 weeks and recruits around 600 men annually. It combines physical exercise with health education. The program promotes health behavior change through male interactions and positive role modelling. Key strategies of the program include content and discussions that encourage men to reflect on and challenge traditional masculine norms that contribute to negative health outcomes. Barriers to preventative health care such as the stigma associated with mental help are challenged. Men are encouraged to openly discuss health concerns and provided with strategies for seeking physical and mental health care in an effort to counter emotional restriction, dysfunctional aspects of self-reliance and reluctance to seek help. Similarly, men are encouraged to see their general health care practitioner for a routine physical health check. Sessions are delivered by invited guests from local health organisations and peak bodies on topics such as cancer screening and heart health to increase health literacy. The inclusion of lived experience speakers aims to normalise preventative health care for men. The physical exercise component is facilitated by exercise physiologists. Men exercise at their own level and pace. They are encouraged to support each other, and competitiveness is not condoned. In the final session men are provided with information on local resources to continue their health promotion endeavours. The program is exclusive to men who reside or work in western metropolitan Melbourne and regional west of Victoria, Australia and is delivered at community facilities in eight local government areas. The majority of men who attend the program reside within 10 postal areas; six are ranked in the bottom 20% of the Socioeconomic Indexes for Areas (SEIFA) [39] for relative socioeconomic advantage and disadvantage in Victoria. The program aims to be inclusive of men living with chronic health conditions. To facilitate safe participation and allocation to one of three exercise intensity levels, men’s health-risk status is assessed using their self-reported medical history prior to the program. Risk is assessed according to the presence of cardiovascular disease or cardiovascular risk factors, pulmonary disease, metabolic disease and musculoskeletal injury. In the year of this study, 22% of program participants were classified by SOTW facilitators as low risk, 50% as medium risk, and 28% as high risk. A full description of the SOTW program and participant characteristics can be found in Vassallo et al. [17]

### Participants

Questionnaire responses were obtained from a sample of men enrolled in the SOTW 2019 program who chose to participate in a study about men’s health. There were 295 participants that were matched with pre-intervention (week one) and post-intervention data (week 10). SOTW participants had sufficient English to engage in the program; there were no further inclusion or exclusion criteria for participation in the research.

### Materials

#### Demographic questionnaire

A demographic questionnaire asked for occupation, age, postal code, and country of origin.

#### Health self-efficacy

The Self-Rated Abilities for Health Practices (SRAHP) [40] formed the basis of the health self-efficacy measure, with additional items from the Self-Efficacy in Seeking Mental Health Care (SE-SMHC) [41] questionnaire to incorporate mental health related items. Several items were removed from both questionnaires to reduce the demand on participants (e.g., brushing your teeth). The final health self-efficacy scale consisted of 20 items and covered four areas of health aligned with SOTW program goals and content (nutrition, healthcare-seeking, psychological help-seeking, and exercise). A ten-point visual-analogue scale was used with three anchor points, not confident (1) somewhat confident (5) and very confident (10), representing the respondent’s confidence in exercising control over a particular behavior. Item scores were summed; higher scores indicated a higher perceived capacity to engage in health promoting behavior. Example questions included “Clearly tell my doctor what aspects of my health are worrying me” and “Overcome any embarrassment I may have about seeking help for my mental health”. A principal components analysis was performed to examine the factor structure. It’s essential to note that while the eigenvalue greater than one rule is popular in some disciplines such as marketing, it can lead to over-extraction, resulting in less parsimonious theories [42]. In this study, an eigenvalue criterion was set at > 0.95, diverging slightly from the often-used eigenvalue > 1 rule to reflect a more conservative approach based on Patil et al.‘s [42] recommendations. A significant Kaiser-Meyer-Olkin and Bartlett’s Test (*p* =.001) suggested the emergence of four distinct factors: exercise, nutrition, healthcare-seeking, and psychological help-seeking. Visual inspection of the scree plot was consistent with a four-factor solution and the pattern-matrix provided a conceptually meaningful loading of items within each component. Reliability testing demonstrated a robust reliability; Cronbach’s alpha ranged from 0.80 to 0.92.

#### Conformity to masculine norms

The Conformity to Masculine Norms Inventory-22 (CMNI-22) [43] was used to measure the degree to which men endorse or conform to the attitudes, behaviors and beliefs associated with traditional western masculine stereotypes. The scale is a shortened version of the original scale developed by Mahalik et al. [6] The self-reported scale consists of 22 questions using Likert-type responses ranging from 0 (“Strongly disagree”) to 3 (“Strongly agree”). The CMNI-22 has 11 subdomains. Questions corresponding to disdain for homosexuals were removed due to the concern by program facilitators that participants would find responding to these questions uncomfortable. The remaining subdomains (with pre- and post-test Chronbach’s alpha reliability statistics shown in parenthesis) were emotional control (α = 0.78; α = 0.83), playboy (α = 0.75; α = 0.84), winning (α = 0.42; α = 0.63), self-reliance (α = 0.60; α = 0.66), pursuit of status (α = 0.43; α = 0.58), risk-taking (α = 0.38; α = 0.38), power over women (α = 0.08; α = 0.34), primacy of work (α = 0.20; α = 0.43), dominance (α = 0.61; α = 0.63) and violence (α = 0.36; α = 0.25). Higher scores indicate a higher adherence to traditional masculine norms. Some example questions include “I should be in charge” and “It bothers me to ask for help”. The inconsistent and predominantly inadequate reliability of the subscales precluded analysis of the relationship between specific aspects of masculinity and health self-efficacy. Therefore, the decision to use the global masculinity measure was made. Item scores were added to compute a total conformity score that was used at pre- and post-program. Cronbach’s alpha was 0.58 pre-intervention and 0.63 post-intervention. Previous studies using the CMNI-22 found similar reliability (e.g., α = 0.61).^43^ This lower reliability can be attributed to the multidimensionality of the scale and potential variation in respondents’ conformity to different aspects of masculinity. For instance, an individual’s conformity to emotional control was found to differ from their alignment with norms related to risk-taking or the playboy ethos [44]. Similarly, Milner et al. [45] reported CMNI-22 subscale reliability scores ranged from a low of α = 0.44 (pursuit of status) to a high of α = 0.81 (playboy). Even though it is a widely used measure of masculinity, similar reliability was encountered when the short form was originally developed; its global indicator’s alpha estimate was 0.67 [43], marginally nearing acceptable thresholds. The removal of two items in the current study likely further contributed to the levels reported here.

#### Psychological distress

The Kessler Psychological Distress Scale (K10) [46] was used to measure psychological distress. The self-reported scale consists of 10 items using Likert-type responses from 1 (“None of the time”) to 5 (“All of the time”) asking how often respondents have felt over the last 30 days. Questions relate to symptoms of anxiety and depression. Item scores are summed to form a single variable of psychological distress and higher scores indicate higher psychological distress. Example questions include “Did you feel hopeless?” and “Did you feel tired out for no good reason?” Reliability analysis indicated Cronbach’s alpha was 0.93 at pre-program and 0.92 at post-program.

#### Health behavior

The health behavior measure, developed in collaboration with the Western Bulldogs Community Foundation, included four key indicators to assess health behavior change relevant to program goals including healthcare-seeking, psychological help-seeking, physical activity, and community activity engagement. Three direct behavioral questions were asked to ascertain participants’ engagement in positive health behaviours. The first two questions were asked to determine community and physical and mental healthcare engagement, “In the last month, have you visited any health services?” and “In the last month, have you been involved in any organized community activities?” A list of possible services or activities was provided as well as the option to add “other” responses. Some example items were “a counsellor or psychologist,” “general practitioner,” “Heart Foundation walking group” and “activities run by local community centers”. A score of one was allocated to each item selected. The final question derived from the literature [47] asked how many days per week the respondent exercised for more than 30 min a day with scores ranging from 0 (“0 days a week”) to 7 (“7 days a week”). Scores from the three items were summed to form a health behavior variable; higher scores indicate higher engagement with health-specific activities and exercise.

### Procedure

Ethics approval was granted prior to the research by the Victoria University Human Research Ethics Committee. The SOTW provided an opportunity to test a model of health behavior change in an established health program, thus enhancing the ecological validity and practical implications of the findings. The program was designed and delivered by the Western Bulldogs Football club community services arm in collaboration with local government and community health services. Research access was facilitated by SOTW coordinators and data collection took place at SOTW sites. The researchers collaborated with program staff in choosing questionnaire items to achieve a balance between the needs of the research for measurement validity and concerns over intrusion and the burden of participation placed on the men. The first author was responsible for data collection and attended each location at the first session (week 1), during the program registration and orientation component, and last session (week 10) of the SOTW program in 2019. During these sessions, men were requested to participate in the survey and provided with an information statement explaining the scope and purpose of their participation, an informed consent form, and the paper-based survey. Participants were invited to clarify any concerns before completing the survey. Participation in the program was voluntary and not contingent on participation in the research. Fifteen minutes was allocated during the session for survey completion, which took place in a group setting of an average of approximately 40 men in each location.

### Analytical plan

#### Preliminary analyses

Descriptive statistics and preliminary hypotheses were conducted using SPSS (version 22.0). Little’s test was conducted to determine whether missing items on constructs were missing at random or systematic. Data was missing at random. For measures with 80% or greater completion of a scale, missing values were replaced with the participant’s mean score derived from the available items on each variable. The average number of missing values per item was 4.3 or 1.46% and 61% of items had five or less missing values. Normality was tested for all variables at both time points and fell within acceptable ranges of + or − 3 which indicates the data was normally distributed. Means and standard deviations from these variables are presented in Table 1. To assess whether significant change occurred, a within-subjects analysis of variance was conducted on pre-test and post-test data. GPower was used to determine adequate power of 0.95 for a two-tailed dependant *t*-test (Faul et al., 2007) [48]. At 0.05 significance level, the report suggested that a sample size of 45 was needed for adequate power.

#### Primary analyses

Examining the changes between pre-intervention and post-intervention are necessary to assess mechanisms of change [24]. To test the mediation hypothesis, we used residualized change score mediation analysis (with bias-correction 95% CIs 5000 bootstrapped samples) rather than other longitudinal approaches because residualized change scores adjust for baseline scores and correlations between measurements [49], while making fewer assumptions about the stability of measures across time compared to raw difference scores [50]. Residualised change score models provide a middle ground when considering the different assumptions of the difference score and ANVOCA models [51]. It has also been suggested that in non-randomized designs, it is preferable to ANCOVA models in the absence of measurement of a robust set of potentially confounding variables [50]. 

The Method for Behavioral, Educational, and Social Sciences (MBESS) package in R Studio was used to conduct the analyses and to produce effect size measures for each mediation model [52]. The outcome variables were the residualized change in both health behavior and psychological distress The predictor was the residualized change score in conformity to masculine norms and the mediator was residualized change in health self-efficacy. Power for the main hypothesis was determined by *a* and *b* path coefficients reported in previous research [53]. Effect sizes for masculine norms, health self-efficacy and health behaviour were from Boman and Walker [23] and Sheeran et al. [14] to evaluate the sample size needed. The number of observations needed for adequate power was 183 and thus the study’s sample was adequate. Mahalanobis distance was used to determine whether there were multivariate outliers (*p* <.001). There were no outliers observed. Linearity was assessed using scatter plots and on face-value were found to be acceptable. The traditional four step requirements for mediation were not assessed as contemporary research suggests these steps carry little meaning (significant direct and total effects) and are no longer a prerequisite to conducting mediation [54]. 

## Results

### Demographics

The average age of participants was 53.34 (*SD* = 14.37). The majority were born in Australia (*n* = 205; 69%). There were 18% (*n* = 52) who originated from Europe, 10% (*n* = 29) from Asia, and 3% (*n* = 9) elsewhere. 22% (*n* = 65) of participants spoke a language other than English at home, 15% (*n* = 44) were receiving some form of disability support, and the primary occupation was retired (*n* = 44). Of the 202 participants who provided postcode information, 36.14% resided in the lowest quintile (i.e. most disadvantaged area) on the index for relative socio-economic disadvantage in Australia. The average baseline score on the CMNI-22 was 21.26 (total possible score = 60). We compared our sample to the older cohort (*N* = 2894; mean age = 51.87) of a national Australian study on male health (mean CMNI = 26.3; total possible score = 66). Our participants, on average, scored lower (35.43% versus 39.85%) than the comparative cohort. This is not unexpected given our sample were slightly older and had chosen to enrol in, and subsequently completed, a health promotion program.

### Program changes

The changes over the course of the SOTW program in health self-efficacy, health behaviour, psychological distress, and conformity to masculine norms were tested. Analysis of variance tests demonstrated a statistically significant increase for health self-efficacy and health behavior and a statistically significant decrease for psychological distress and conformity to masculine norms (see Table 1).

**Table 1:** Changes in health behavior, health self-efficacy, psychological distress and conformity to masculine norms

Variable	T1 M (SD)	T2 M (SD)	df	F	*p*	η_*p*_^2^
Health Behavior	6.19 (*2.82*)	7.25 (*2.64*)	294	55.83	0.001	0.16
Health Self-Efficacy	143.52(*27.60*)	158.47 (*25.87*)	286	103.33	0.001	0.27
Psychological Distress	18.47 (*7.69*)	17.19 (*7.10*)	283	14.61	0.001	0.05
CMN	21.26 (*5.14*)	20.44 (*5.24*)	268	10.67	0.001	0.04

Note. T1 = pre-program (week 1). T2 = post-program (week 10). *M* = mean. *SD* = standard deviation. η_p_^2^ = partial eta square. CMN = conformity to masculine norms

Prior to conducting mediation analysis, simple bivariate relationships were examined and can be seen in Table 2. As expected, there was a significant positive relationship between health self-efficacy and health behavior, a significant negative relationship between health self-efficacy and conformity to masculine norms and psychological distress, and a significant positive relationship between conformity to masculine norms and psychological distress. These relationships were consistent at pre-intervention and post-intervention.

**Table 2:** Correlates of Pre-Intervention and Post-Intervention measurements

Measures	1	2	3	4	5	6	7	8
1. T1 Health Behavior	-	− 0.10	0.30**	− 0.40	0.65**	0.10	0.11	0.04
2. T1 CMN		-	− 0.19**	0.14**	− 0.21**	0.69**	− 0.22**	0.14*
3. T1 Health Self-Efficacy			-	− 0.46**	0.25**	− 0.13*	0.58**	− 0.36**
4. T1 Psychological Distress				-	0.02	0.06	− 0.34**	0.72**
5. T2 Health Behavior					-	− 0.21**	0.19**	0.02
6. T2 CMN						-	− 0.29**	0.17*
7. T2 Health Self-Efficacy							-	− 0.44**
8. T2 Psychological Distress								-
Note. *statistically significant at the 0.05 level. **statistically significant at the 0.001 level. T1 = pre-program (week 1). T2 = post-program (week 10). CMN = Conformity to Masculine Norms.								

### Mediation analysis

The following analyses refer to the mechanisms of change observed in gender-sensitised health promotion and whether changes in health self-efficacy would mediate the relationship between changes in conformity to masculine norms and changes in health outcomes.

**Fig. 1:**
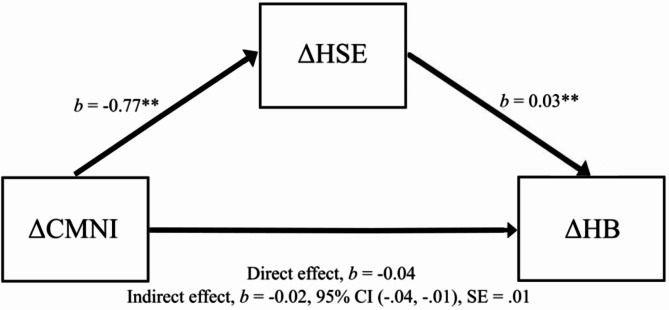
Model predicting health behavior change via a health self-efficacy– conformity to masculine norms mediated pathway. **Legend:** ∆CMNI = Conformity to Masculine Norms residualized change scores. ∆HSE = Health Self-Efficacy residualized change scores. ∆HB = Health Behavior residualized change scores. Unstandardized coefficients presented of the *a*,* b* and *c’* prime paths. * *p* <.05. ** *p* <.001. *b* = unstandardized coefficient. SE = standard error. CI = Confidence Intervals for bootstrap methods between lower level and upper level

A residualized change score mediation analysis with changes in health behavior as the outcome found the indirect effect of changes in health self-efficacy was statistically significant (see Fig. 1). The unstandardized coefficients for the a, b, and c’ prime path can be seen in Fig. 1. There was a significant indirect effect and a non-significant direct effect, indicating a fully mediated pathway from changes in conformity to masculine norms to changes in health behaviour via changes in health self-efficacy. The prediction of changes in health behaviour from changes in conformity to masculine norms alone was not significant, *b* = -0.02, *t*(264) = 0.57, *p* =.463, 95% CI = [-0.07, 0.03]. The mediation effect size using Kappa-squared ( *κ*^2^ = 0.06) indicates a small effect size [55]. 

**Fig. 2:**
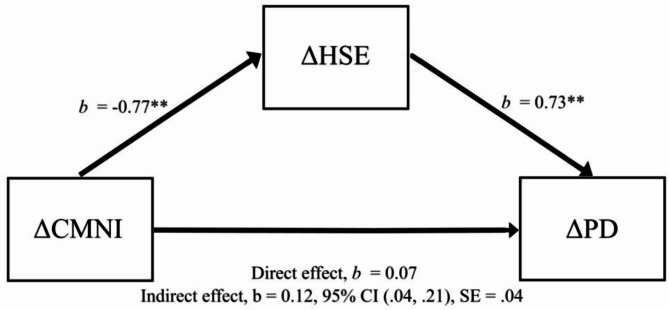
Model Predicting Psychological Distress Change via a Health Self-Efficacy– Conformity to Masculine Norms Mediated Pathway. **Legend:** ∆CMNI = Conformity to Masculine Norms residualized change scores. ∆HSE = Health Self-Efficacy residualized change scores. ∆PD = Psychological Distress residualized change scores. Unstandardized coefficients presented of the *a*,* b* and *c’* prime paths. * *p* <.05. ** *p* <.001. *b* = unstandardized coefficient. SE = standard error. CI = Confidence Intervals for bootstrap methods between lower level and upper level

The second residualized change score mediation analysis with changes in psychological distress as the outcome found the indirect effect of changes in health self-efficacy to be statistically significant (see Fig. 2). The unstandardized coefficients for the a, b, and *c’* prime path can be seen in Fig. 2. There was a significant indirect effect and a non-significant direct effect, indicating a fully mediated pathway from changes in conformity to masculine norms to changes in psychological distress via changes in health self-efficacy. The prediction of changes in psychological distress from changes in conformity to masculine norms alone was not significant, *b* = 0.08, *t*(264) = 0.95, *p* =.340, 95% CI [0.03, 0.36]. The mediation effect size for Kappa-squared (*κ*^2^ = 0.09) indicates a medium effect size [55]. 

## Discussion

The aim of the study was to examine the mechanisms of men’s health behavior change in a sample of men participating in the 2019 SOTW program. The findings showed health self-efficacy, health behavior, adherence to masculine norms and psychological distress improved over the ten weeks and were associated in complex ways. The changes in men’s outcomes from pre-program to post-program were examined to investigate the mechanisms of change observed over the course of the program. Changes in health self-efficacy fully mediated the relationship between changes in adherence to masculine norms and changes in psychological distress. Although there was no significant bivariate relationship between changes in conformity to masculine norms and health behavior, a mediation analysis showed a mediated pathway from changes in conformity to masculine norms to changes in health self-efficacy to changes in health behavior. The absence of a relationship between conformity to masculine norms and health behavior is not unexpected, given the mediated pathway includes a negative relationship followed by a positive relationship.

This study was the first to longitudinally examine the mechanism of men’s health behavior change within the setting of a gender-sensitised intervention. The findings suggest that increasing flexibility towards traditional masculine norms to increase health self-efficacy is an effective approach for men’s health promotion. In particular, gender-sensitised health promotion may be an optimal approach for men to gain this flexibility [56, 57, 58]. The mediation effects were similar to previous research showing health self-efficacy partially mediated the association between masculine norms and health outcomes in a community sample using a cross-sectional design [16], although, the findings from the current study observed larger indirect effects, smaller direct effects and full mediation. The findings contribute to research examining the effects of adherence to traditional masculine norms on men’s physical and psychological health [7, 8]. 

Improvements in health are possible when men successfully challenge traditional masculine norms, gaining flexibility to engage in mental and physical health promotion. This study found full mediation in models predicting health behavior and psychological distress and indicated the changes in traditional masculine norms were associated with improved health self-efficacy, which was then associated with improved health outcomes. When conformity is high, there may be little flexibility to engage in health-promoting behaviors, even when men recognize change is necessary. For instance, McKenzie et al. [59] suggest men refrain from seeking emotionally supportive relationships due to rigid adherence to masculine norms of self-reliance and emotional inexpressiveness. When men gain flexibility around these norms, they may then feel less social pressure and enact pathways to health promotion [12, 16, 60, 61]. 

The findings also contribute to the body of research demonstrating the effectiveness of gender-sensitised health promotion where improvements in health behavior, health self-efficacy, psychological distress and conformity to traditional masculine norms were observed [17, 31, 56]. The findings support the underlying premise of gender-sensitised health promotion where men participating in these spaces are encouraged to overcome the limitations related to strict adherence to traditional masculine norms, in turn developing greater health self-efficacy beliefs [37]. The community setting of a football club, male-only participation, provision of role-models and an environment that supports flexible views of masculinity likely foster a sense of safety that encourages an open dialogue [17]. This space provides an opportunity for learning from similar men who have successfully negotiated traditional masculine norms without experiencing pressure to adhere to gender-socialized stereotypes [19, 62, 63]. The current study utilised a sample of mostly older men, many of whom had chronic lifestyle related health conditions. Understanding the association between conformity to masculine norms and health behaviour has important implications for populations with entrenched poor health behavior and those where stereotypical masculinity presents barriers to health seeking behaviors.

Robertson et al. [62] explain safe spaces are facilitated by providing community-based alternatives to formal and clinical settings that may deter men from participation. The sense of safety leads to trust and the opportunity to practice and reflect on health, developing self-efficacy through meaningful social interactions [64]. Developing meaningful bonds among participants may further encourage health related discourse, which helps to refashion masculine beliefs and illuminates how they affect health promotion [65, 66]. The community setting and the supportive group atmosphere plays an integral role in health self-efficacy development that contributes to an increased confidence to initiate and practice health behaviors in ways that are more acceptable for men [13]. 

There are several limitations in this study. Firstly, while this study’s mediation analyses showed significant effects, it is crucial to note that the effect sizes were small to moderate (κ2 = 0.06 for health behavior and κ2 = 0.09 for psychological distress). These modest effect sizes and potential for Type 1 error suggest that while the masculinity-self-efficacy mechanism appears to play a role in health behavior change, its practical significance should not be overstated without further replication and research. While significant improvements in health outcomes were observed, the small to moderate mediation effects suggest that the masculinity– self-efficacy mechanism may be one of several pathways through which gender-sensitised programs influence health outcomes Also, the items used to measure health behavior had limited psychometric properties; it included items developed in collaboration with the SOTW coordinators to align with program goals with a focus on recreational and physical activities. The reliance on self-report measures, which are subject to responder bias, is a further limitation of this study. A recent review exploring the correlations between conformity to masculine norms and health outcomes found there was no relationship with regard to outcomes specific to physical activity [7]. This likely accounts for the lack of direct association observed between masculine norms and health behavior in the current study compared to previous research [16]. Measurements including a broader range of health behaviours, including those specific to healthcare access, would provide a better understanding of the relationship between masculine norms and health behavior. Ideally, objective measurement of anticipated health outcomes associated with the targeted health behaviour change, such as improvement in cardiovascular fitness, should be included in future research. This would overcome the bias associated with self-report measures and add further support to a gender-sensitized approach to men’s health programs.

Additionally, previous research has illustrated that aggregating masculinity in terms of its implication to health is outdated and suggests targeting key aspects of masculinity that contribute to negative health outcomes is a more innovative approach to men’s health [8]. The inadequate reliability of the conformity to masculine norms subscales was a further limitation of the current study. We were particularly mindful of not overburdening participants and restricted the survey length accordingly, which most likely contributed to the poor reliability. Future research should analyse specific masculine subdomains as predictors and compare their direct and indirect effects along with other factors such as social support in more complex models. In similar settings to the current study, where survey length is a concern, it may be advisable to use longer form measures of fewer sub-domains, selecting only those aspects of masculinity that are theoretically relevant or have previously been found to negatively predict health outcomes, such as self-reliance, risk taking and power over women [8, 9]. Future research should also address the challenges involved with research designs in real-world settings. Large scale follow-up analysis using three waves of data would provide more robust causal inference of this mechanism of men’s health promotion. A third wave of data collection, ideally six months or longer post-program, would allow the maintenance of observed changes in conformity to masculine norms, health self-efficacy, health behaviours and health outcomes to be assessed.

Although the current research is correlational in nature and lacks a control group, it has included longitudinal change, which adds weight to the body of research on how conformity to masculine norms impact health. Future research that qualitatively explores the shared experiences of negotiating masculinity to improve health will extend the current understanding and further illustrate the unique processes contributing to increases in health self-efficacy in gender-sensitised contexts. Nevertheless, this is the first study to examine the mechanisms of men’s health behavior change in men participating in a large-scale, real-world gender-sensitised health behavior intervention. Large effect sizes for health improvements were observed and the full mediation effect indicates changes are likely due to a masculinity-self-efficacy mechanism wherein greater flexibility around masculine norms lead to increases in health self-efficacy with subsequent health benefits. Evidence of cross-cultural validity mounts with programs effective in Europe [31] and Canada [56]. This ever-increasing diversification of settings illustrates gender-sensitised health promotion likely applies to a variety of male-orientated spaces and initiatives would benefit by adapting masculine-specific behavior change techniques to take full advantage of gender-sensitised health promotion.

## Data Availability

The datasets generated and analysed during the current study are not publicly available due to the sharing of data not being included in information provided to participants at the time of obtaining informed consent but are available from the corresponding author on reasonable request.

## Abbreviations

SOTW: Sons of the West
SEIFA: Socioeconomic Indexes for Areas
SRAHP: Self-Rated Abilities for Health Practices
SE-SMHC: Self-Efficacy in Seeking Mental Health Care
CMNI-22: Conformity to Masculine Norms Inventory-22
K10: Kessler Psychological Distress Scale
MBESS: Method for Behavioral, Educational, and Social Sciences (package in R Studio)
T1: Pre-program
T2: Post-program
